# Ill. Charitable Eye and Ear Infirmary

**Published:** 1875-06

**Authors:** E. L. Holmes


### Clinics.

### ILLINOIS CHARITABLE EYE AND EAR INFIRMARY.

*Clinical Lectures by* Dr. E. L. Holmes.

### CONJUNCTIVITIS OF NEW BORN INFANTS. MALIGNANT DISEASES OF THE EYE.

The two cases of conjunctivitis before us to-day present good examples of the results of the affection when neglected. Recalling to mind the nine other patients which you have seen the past few months, you will conclude that an institution like this seldom presents an example of the disease in its primary stage. You have not seen at the clinic an infant which was less than three weeks old. Two have been more than two months old.

You as physicians will be called upon to treat these patients in the early stages of the inflammation. It is your duty to know all that can be known regarding the best method of treatment. You have witnessed most sad examples of neglect; for two patients have been brought here blind, with both corneæ sloughed.

There is scarcely any disease of the eye so easily controlled as this if the proper remedies are early applied. In the outset I must urge you to make a distinction between the treatment required early in the course of the disease, and that which you have witnessed here in the later stages.

I think in nearly all cases the affection is a simple catarrhal inflammation. The conjunctiva in infancy seems especially susceptible to the influences which produce catarrh. Conjunctivitis is very liable at this age to assume a purulent form. Statistics, based on the condition of the mother, as regards the presence of muco-purulent discharge from the vagina during labor, scarcely indicate that such discharges generally produce the conjunctivitis.

The symptoms of the disease vary in degree. There is generally at first a slight redness, with an increase of tears. A very rapid and extensive swelling is apt to follow, with an excessive quantity of thick purulent discharge. The swelling may be so great that it is almost impossible to evert the lids. Occasionally the swollen mucous membrane is found to be covered with a thin pellicle of partially organized lymph. This condition has been termed membranous conjunctivitis. This, however, is far from being diphtheritic inflammation, which is characterized by an exudation of organizable lymph *into* the substance of the conjunctiva, as well as on its surface. This form of diphtheria is most dangerous to the eye, but is fortunately rare in this country.

The treatment of conjunctivitis neonætorum is upon the whole very simple. In all cases it is of the utmost importance to keep the eyes scrupulously clean. To accomplish this requires some skill in turning the lids. Some authors recommend the use of very weak astringent solutions, thrown every hour or even oftener, from a syringe, between the lids. Unless the nurse has experience, there is danger of injuring the eye with the instrument; there is danger also, without care, that some of the discharge may be thrown into the eyes of those holding the patient. I apprehend the great difficulty you will meet in the thinly settled portions of the country will be the want of skilful nurses to follow your directions, if you trust to weak solutions often applied.

In the early stages of the disease, especially when the symptoms are slight, Alum gr. ij—v to the ounce; Zinc Sulph. gr. j—ij; Cupri Sulph. gr. j, are perhaps the astringents most commonly used.

But it not unfrequently happens that in spite of this treatment the inflammation assumes a most violent type. The swelling of the conjunctiva, œdemæ of the lids, and amount of the discharge, may become enormous. The cornea sometimes sloughs in two days, or may remain intact for weeks.

The cases you have seen here have, without exception, been very grave. In three of them the cornea of one eye, in two the corneæ of both eyes, had already been destroyed. In three more the corneæ had become more or less ulcerated. In all, the lids were much swollen, and the conjunctiva roughened to an extreme degree.

You have so frequently witnessed the method of treating these cases in the clinic that I need not dwell upon it now. After exposing the mucous membrane as extensively as possible, I gently soak up the pus and moisture with a soft bit of linen, and apply evenly but carefully over the whole surface a solution of Arg. Nit. ℈ j—ij to the ounce of water, with quite a large camel's hair pencil, *well moistened* but not *dripping* with the solution. The "granulations," without exception, you have seen disappear, and the cornea left normally clear, where there were not previously opacities or ulcerations. You have very often expressed surprise that so strong a solution should produce apparently no pain. I can rely on your testimony that, except while the babes' heads were held and the lids opened, the infants have never cried. They have day after day been carried from the clinic room after the treatment without uttering a sign of pain.

In case the hypertrophy of the conjunctiva is excessive to a remarkable degree, as in two cases, I am in the habit, immediately after the cautery, of excising a thin fold the whole length of the lid, as near the duplicature as possible. With such great swelling the piece excised comprises, in reality, but a minute portion of the true tissue. The local bleeding seems to aid in giving relief. Recent authorities recommend in extreme cases the elongation of the palpebral fissure at the external angle. This little operation relieves tension and causes considerable local depletion.

I cannot leave this subject without again impressing upon you most earnestly the importance of not confounding the treatment you have observed at the infirmary with that which is appropriate for the ordinary cases you will meet as physicians in the first few days of the infant's life. Moreover, remember the rule I hold of utmost importance, that the stronger the solution of nitrate of silver you use, the less of it there should be on the brush with which it is applied. Without these two precautions you are in danger of doing more harm than good.

MALIGNANT DISEASES.

From the number of cancerous growths which you have recently observed here, you must not infer that they always occur so frequently at the clinic. The large number this winter is accidental.

We have two patients which I requested to be at the Infirmary to-day, presenting examples of sarcomatous tumors of the choroid, recurring in the orbit about six months after extirpation of the globe by other surgeons. I must refer you to the last clinic for a somewhat more extended account of choroidal sarcoma. In one case the orbit is completely filled. No surgical interference I believe is warrantable. In the other case, the tumor seems to be attached to the free ends of the muscles in some way, for the round tumor rotates quite freely with the motions of the other eye. Moreover, the tip of the finger can be pressed some distance between the orbit and tumor on every side. I am disposed to advise the removal of the growth. After the operation you perceive that the orbit is left apparently free from diseased tissue. It has been my experience, as, I believe, it is the general experience, that choroidal sarcoma, after the removal of the globe, is not prone to reappear in the orbit, but rather in some internal organ. This tumor is composed of two portions, one a soft black tissue, the other a hard white schirrus. I apprehend this last fact renders the prognosis bad as regards the return in the orbit.

You observe in this other patient, seventy years of age, an example of melanotic tumor of the conjunctiva. He states that for many years there was a small black spot a few lines from the cornea in the upper and inner quarter of the ocular conjunctiva. About two years ago it began to enlarge, and was finally removed by his physician as a small pedunculated tumor.

We now find, five months after the operation, a tumor about three-eighths of an inch in diameter and somewhat pedunculated. It is not apparently attached to the sclerotic, for it can be freely moved over this membrane. I think the patient's comfort and prospect of prolonged life will be enhanced by the removal of the tumor, with quite an extensive portion of the conjunctiva and subconjunctival tissue.

The contraction produced by the cicatrization of this extensive wound, I have informed the patient, will undoubtedly cause the globe to be drawn permanently upward and inward, and consequently restrict the movements of the eye.

